# Supplementary material for: Secular Trends in Physical Fitness of Peruvian Children Living at High-Altitude
Source: Int J Environ Res Public Health. 2023 Feb 12;20(4):3236. doi: 10.3390/ijerph20043236 (PMC9958918; doi:10.3390/ijerph20043236)
Supplement: Supplementary file 1 [file ijerph-20-03236-s001.zip › ijerph-2146613-supplementary.pdf]

Table S1. Mean differences (adjusted means ± standard errors, differences (diff) and their corresponding 95% confidence intervals (95%CI)), and two-factor ANCOVA model, adjusted for height and weight, main results for each physical fitness test in girls between 2009 and 2019 cohorts.

Girls																
	**Handgrip (kgf)**				**Standing Long Jump** (cm)				**Sit and reach (cm)**				**Shuttle-run (s)**			
**Age (y)**	**2009**	**2019**	**Diff.**	**95%CI**	**2009**	**2019**	**Diff.**	**95%CI**	**2009**	**2019**	**Diff.**	**95%CI**	**2009**	**2019**	**Diff.**	**95%CI**
6	9.1±0.5	9.5±0.4	0.4^{ns}	-0.63; 1.50	101.8±3.2	88.6±2.9	-13.2**	-20.3; -6.14	19.6±0.9	23.1±0.8	3.5**	1.41; 5.43	28.5±0.5	31.2±0.4	2.7**	1.68; 3.77
7	9.1±0.4	9.7±0.3	0.6^{ns}	-0.28; 1.49	97.2±2.6	94.6±2.2	-2.6^{ns}	-8.48; 3.27	20.6±0.7	22.2±0.6	1.6*	0.02; 3.36	29.2±0.4	29.9±0.3	0.7^{ns}	-0.14; 1.59
8	9.1±0.4	9.7±0.3	0.6^{ns}	-0.39; 1.47	104.9±2.5	94.6±2.0	-10.3**	-16.5; -4.21	19.8±0.7	22.2±0.6	2.4**	0.58; 4.08	29.2±0.4	29.1±0.3	-0.1^{ns}	-0.95; 0.86
9	9.6±0.3	10.1±0.3	0.5^{ns}	-0.34; 1.34	104.3±2.1	97.4±1.9	-6.9*	-12.4; -1.35	20.3±0.6	22.2±0.5	1.9*	0.29; 3.44	28.9±0.3	28.8±0.3	-0.1^{ns}	-0.99; 0.64
10	10.7±0.3	10.5±0.3	0.2^{ns}	-0.98; 0.63	106.8±2.1	101.5±2.0	-5.3*	-10.7; -0.01	20.7±0.6	22.9±0.6	2.2**	0.75; 3.78	29.1±0.3	28.0±0.3	-1.1**	-1.87; -0.29
11	12.6±0.3	12.3±0.3	0.3^{ns}	-1.18; 0.41	110.7±2.3	103.9±2.3	-6.8**	-12.0; -1.53	22.1±0.7	23.9±0.7	1.8*	0.37; 3.35	28.1±0.3	27.1±0.3	-1.0**	-1.85; 0.30
Two-Factor ANOVA Model Results																
Age	F = 14.64, p < 0.001, η^2 = 0.085				F = 2.77, p < 0.001, η^2 = 0.017				F = 1.91, p = 0.089, η^2 = 0.012				F = 4.15, p < 0.001, η^2 = 0.026			
Year	F = 1.79, p = 0.181, η^2 = 0.002				F = 36.07, p < 0.001, η^2 = 0.044				F = 39.42, p < 0.001, η^2 = 0.048				F = 0.94, p = 0.333, η^2 = 0.001			
Age-by-Year	F = 1.00, p = 0.418, η^2 = 0.006				F = 1.34, p = 0.246, η^2 = 0.008				F = 0.44, p = 0.823, η^2 = 0.003				F = 8.77, p < 0.001, η^2 = 0.528			

Note: adjusted means for height and weight; ^{ns}=non-statistically significant; *, p<0.05; **, p≤0.01

Table S2. Mean differences (adjusted means ± standard errors, differences (diff) and their corresponding 95% confidence intervals (95%CI)), and two-factor ANCOVA model, adjusted for height and weight, main results for each physical fitness test in boys between 2009 and 2019 cohorts.

**Boys**																
	**Handgrip (kgf)**				**Standing Long Jump (cm)**				**Sit and reach (cm)**				**Shuttle-run (s)**			
**Age (y)**	**2009**	**2019**	**Diff.**	**95%CI**	**2009**	**2019**	**Diff.**	**95%CI**	**2009**	**2019**	**Diff.**	**95%CI**	**2009**	**2019**	**Diff.**	**95%CI**
6	9.1±0.5	9.5±0.4	0.1^{ns}	-0.91; 1.07	103.6±2.7	91.7±2.6	-11.9^{**}	-18.13; -5.64	21.9±0.8	22.4±0.8	0.5^{ns}	-1.42; 2.30	27.5±0.5	29.3±0.4	1.8^{**}	0.74; 2.90
7	9.1±0.4	9.7±0.3	0.3^{ns}	-0.61; 1.23	103.1±2.5	93.9±2.0	-9.2^{**}	-15.07; -3.47	22.3±0.7	22.0±0.6	-0.3^{ns}	-2.04; 1.41	27.6±0.4	29.1±0.3	1.5^{**}	0.47; 2.47
8	9.1±0.4	9.7±0.3	-0.2^{ns}	-1.04; 0.58	106.3±2.0	103.7±1.7	-2.6^{ns}	-7.83; 2.48	21.4±0.6	21.1±0.5	-0.3^{ns}	-1.78; 1.29	28.2±0.3	27.5±0.3	-0.7^{ns}	-1.59; 0.19
9	9.6±0.3	10.1±0.3	-0.1^{ns}	-0.83; 0.72	112.4±1.8	111.3±1.7	-1.1^{ns}	-5.98; 3.88	20.7±0.5	20.2±0.5	-0.5^{ns}	-1.97; 0.97	27.7±0.3	27.4±0.3	-0.3^{ns}	-1.23; 0.47
10	10.7±0.3	10.5±0.3	-0.2^{ns}	-1.07; 0.59	118.4±2.0	114.5±2.0	-3.9^{ns}	-9.12; 1.39	19.3±0.6	19.7±0.6	0.4^{ns}	-1.13; 2.01	27.9±0.3	27.4±0.3	-0.5^{ns}	-1.32; 0.49
11	12.6±0.3	12.3±0.3	-0.8^{ns}	-1.67; 0.10	122.2±2.3	119.5±2.3	-2.7^{ns}	-8.31; 2.89	20.6±0.7	20.2±0.7	-0.4^{ns}	-2.01; 1.33	27.4±0.4	26.3±0.4	-1.1^{*}	-2.05; -0.12

Two-Factor ANOVA Model Results				
Age	F = 7.97, p < 0.001, η^2 = 0.049	F = 15.53, p < 0.001, η^2 = 0.091	F = 3.05, p= 0.009, η^2 = 0.019	F = 2.10, p = 0.063, η^2 = 0.013
Year	F = 0.69, p = 0.405, η^2 = 0.001	F = 20.28, p < 0.001, η^2 = 0.025	F = 0.06, p = 0.801, η^2 = 0.000	F = 0.34, p = 0.559, η^2 = 0.004
Age-by-Year	F = 0.66, p = 0.654, η^2 = 0.004	F = 2.16, p = 0.057, η^2 = 0.014	F = 0.25, p = 0.939, η^2 = 0.002	F = 5.65, p < 0001, η^2 = 0.035

Note: adjusted means for height and weight; ^{ns}=non-statistically significant; *, p<0.05; **, p≤0.01

Table S3. Girls' and boys' mean values (± standard deviations) of height, weight and BMIz-score in the two study cohorts.

Age (years)	Girls						Boys					
	2009			2019			2009			2019		
	Height (cm)	Weight (kg)	BMIz-score	Height (cm)	Weight (kg)	BMIz-score	Height (cm)	Weight (kg)	BMIz-score	Height (cm)	Weight (kg)	BMIz-score
6	111.8±4.0	19.5±1.9	0.11±0.79	113.2±3.7	20.8±2.3	0.43±0.88	113.1±4.2	20.0±2.2	0.10±0.77	114.1±5.3	21.1±2.7	0.44±0.95
7	116.9±4.8	21.9±2.9	0.18±0.73	118.1±4.9	22.9±3.7	0.34±0.84	117.8±5.3	22.2±2.6	0.19±0.87	119.2±4.3	22.9±4.0	0.10±1.19
8	124.7±4.9	24.3±3.1	-0.25±0.81	124.6±6.4	26.6±4.5	0.47±0.82	123.7±5.3	24.6±3.9	-0.07±1.02	125.6±6.5	27.2±5.3	0.54±1.03
9	127.5±5.4	26.1±3.8	-0.27±0.84	130.6±5.5	29.2±4.6	0.21±0.81	127.5±5.4	26.1±3.8	0.27±0.86	129.5±5.4	29.1±5.8	0.34±1.14
10	134.0±6.2	29.7±4.5	-0.29±0.92	136.1±6.4	33.2±6.4	0.22±0.97	134.0±6.2	29.7±4.5	0.03±0.82	134.3±6.1	32.8±6.4	0.47±1.06
11	139.6±6.2	34.5±6.6	-0.17±0.86	141.8±5.9	37.8±7.4	0.27±1.01	139.6±6.2	34.5±6.6	1.00±0.72	139.1±6.0	35.8±7.1	0.31±1.09

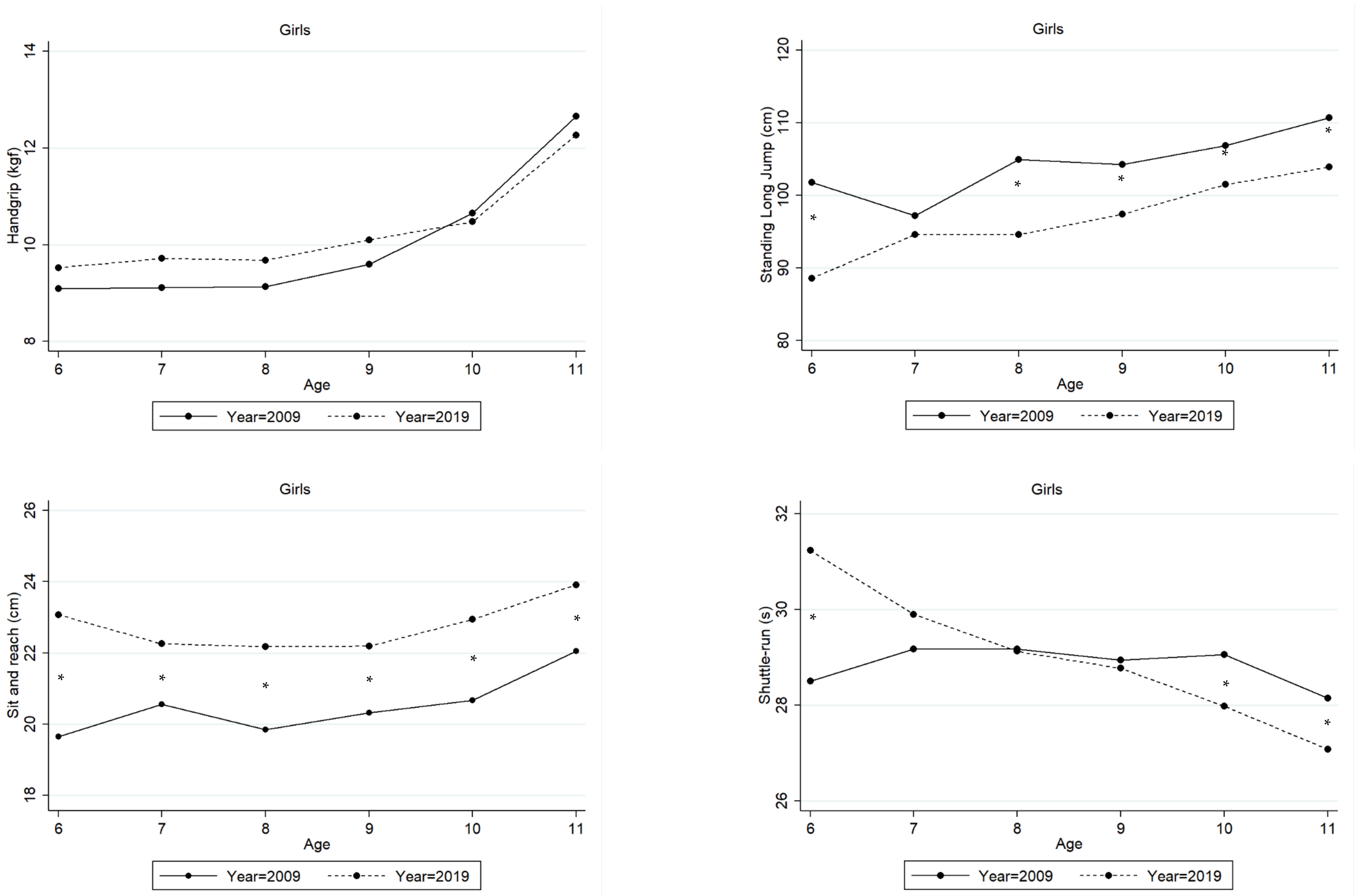

Figure S1. Physical fitness tests' adjusted means in Peruvian girls between 2009 and 2019 cohorts. Results are adjusted for trends in height and weight. Significant differences are marked by *

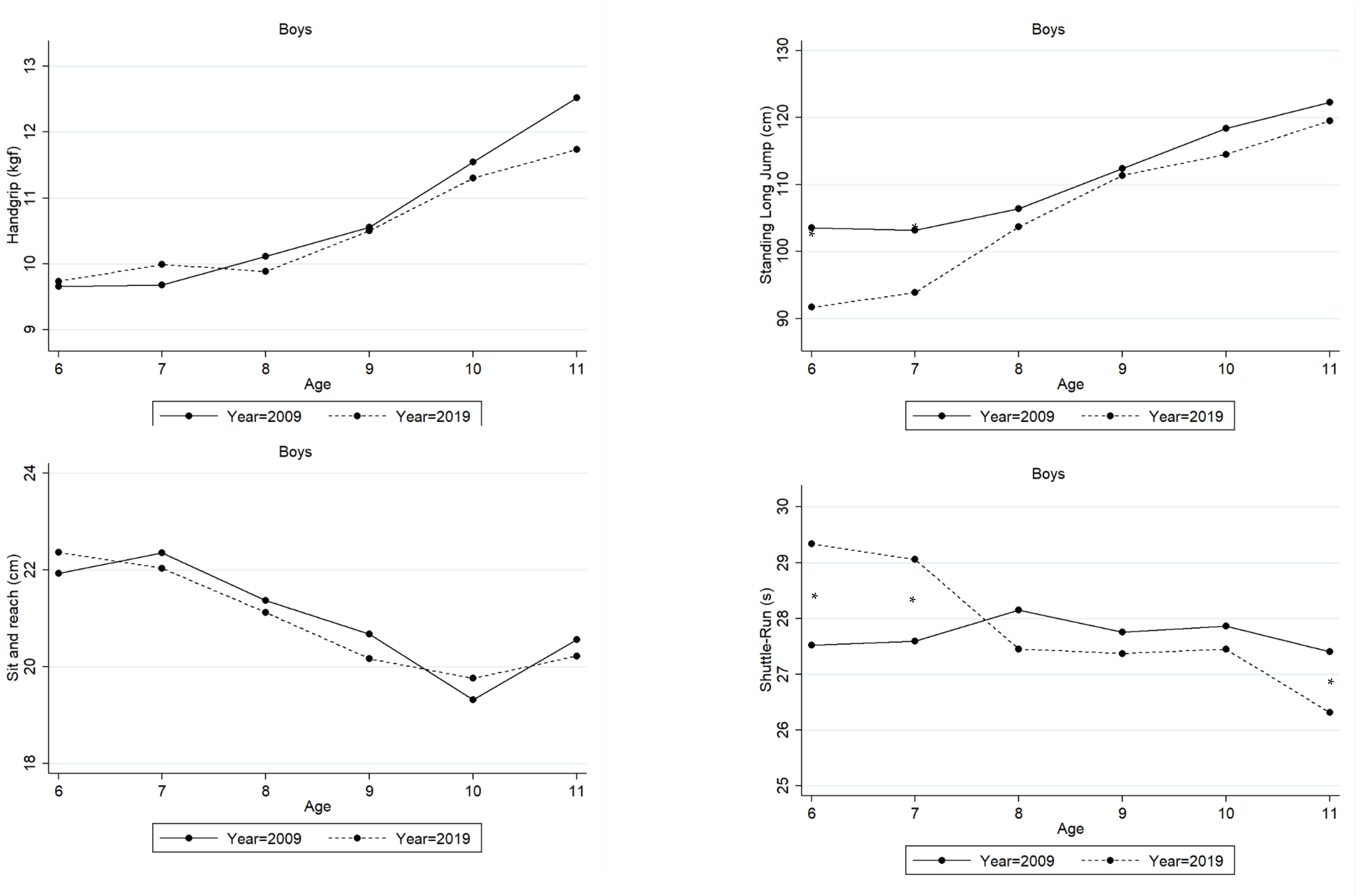

Figure S2. Physical fitness tests' adjusted means in Peruvian boys between 2009 and 2019 cohorts. Results are adjusted for trends in height and weight. Significant differences are marked by *
